# Atypical Presentation of Metformin-associated Lactic Acidosis: A Case Report

**DOI:** 10.5811/cpcem.50572

**Published:** 2026-04-07

**Authors:** Elizabeth Welsch, Jerome Evans, Alexander Yoxall, Anna Culhane

**Affiliations:** * Medical College of Wisconsin, Milwaukee, Wisconsin; † University of Wisconsin, Madison, Wisconsin; ‡ Medical College of Wisconsin, Department of Emergency Medicine, Milwaukee, Wisconsin

**Keywords:** metformin, lactic acidosis, case report

## Abstract

**Introduction:**

Metformin, a first-line type two diabetes medication, is generally considered safe and effective. However, it is rarely associated with life-threatening lactic acidosis. This generally presents in patients with gastrointestinal upset as a primary complaint. It is most common in patients with underlying chronic kidney disease. Prevention of associated mortality requires early diagnosis and intervention with fluids, bicarbonate, vasopressors, and hemodialysis.

**Case Report:**

This is a notable presentation of metformin toxicity, as the 68-year-old male patient presented with an atypical chief complaint of dyspnea and no history of kidney disease. Physical exam was notable for tachypnea and clear breath sounds. Labs revealed anion gap metabolic acidosis from an accumulation of lactic acid and acute renal failure. Other causes of lactic acid metabolic acidosis were considered and ruled out. Nephrology was consulted and pharmacological therapies started as the patient transitioned to the intensive care unit for emergent hemodialysis. He eventually regained renal function and was discharged. His metformin level returned several weeks later significantly elevated.

**Conclusion:**

Emergency physicians should maintain metformin toxicity on the differential for patients on metformin presenting with dyspnea and anion gap metabolic acidosis with elevated serum lactic acid concentrations. Other sources of lactic acidosis should be ruled out, and corrective therapies, including renal replacement therapies, should be started immediately. Additionally, it is essential to determine a patient’s kidney function before being prescribed metformin and to have the patient serially monitored as an outpatient.

## INTRODUCTION

The American Diabetes Association identifies metformin as a safe, first-line drug for the treatment of type two diabetes mellitus, a condition that affects over 34 million people in the United States.^1^,^2^ However, patients with decreased kidney function are at risk of developing metformin toxicity, the most severe form of which causes an elevated anion gap metabolic acidosis known as metformin-associated lactic acidosis, characterized by elevated blood lactate levels (> 5 millimoles per liter [mmol/L]) and decreased blood pH (< than 7.35).^1^,^3^ While rare, metformin-associated lactic acidosis has a mortality rate of up to 50%.^8^ However, early diagnosis and intervention with fluids, bicarbonate, vasopressors, and hemodialysis can lead to more positive outcomes.^3^ For this reason, it is important to consider this diagnosis early in a patient’s presentation to begin prompt treatment.

While metformin-associated lactic acidosis is a rare diagnosis, this case is unique for its presentation with an atypical chief complaint and lack of predisposing risk factors. Prior case reports cite gastrointestinal (GI) symptoms as the patient’s primary complaint.^4^ In this case the patient’s chief concern was shortness of breath, with GI upset only being elicited through thorough history taking. Additionally, the majority of patients in prior case reports had existing renal pathology that predisposed them to metformin-associated lactic acidosis.^5^ Our patient was at his baseline creatinine of 1.03 milligrams per deciliter (mg/dL) (0.70–1.30 mg/dL) three months prior to his presentation.

## CASE REPORT

A 68-year-old male presented to the emergency department (ED) with a chief complaint of dyspnea. On interview, the patient stated he had dyspnea and cough since the day before. He also had an episode of emesis one night prior. This occurred before the shortness of breath. He did not have further emesis and had no abdominal pain. He had not attempted to eat or drink much since the emesis occurred. The patient was blind and was unable to describe his sputum or emesis. He denied any chest pain, orthopnea, dysuria, diarrhea, constipation, fever, chills, or sick contacts. He reported no difficulties with urinating. He denied any alcohol, drug, or tobacco use. He additionally denied any history of asthma, chronic obstructive pulmonary disease, renal disease, or heart failure. Vital signs were as follows: heart rate, 69 beats per minute; blood pressure, 138/58 millimeters of mercury (mm Hg); respiratory rate, 18 breaths per minute; pulse oximetry, 97% on room air; and temperature, 37.0 °C.

The patient had not received care at our facility since 2016; therefore, chart review was limited. He had a past medical history significant for congenital blindness, type 2 diabetes with a hemoglobin A1c of 7.1% from three months prior, hyperlipidemia, hypertension, and venous stasis ulcers. There was no familial or social history on file. The patient’s last primary care visit had been three months prior to his ED presentation. At that time, his metformin dose was increased from 500 mg twice daily to 1000 mg twice daily. He was also started on 20 mg of furosemide daily for bilateral lower extremity lymphedema. He had not had his renal function checked since these medication changes. His blood pressure was noted to be 128/82 mm Hg. He had been taking 12.5 mg of hydrochlorathiazide daily for many years, and this medication had not changed.

On physical exam in the ED, the patient was in marked respiratory distress with tachypnea. His respiratory rate appeared significantly higher than the 18 breaths per minute recorded in the triage note. Pertinently, he had clear breath sounds bilaterally on auscultation. He was normocephalic and atraumatic. He had normal heart sounds with normal rate and rhythm. His abdominal exam was benign, with no distension, guarding, or tenderness. His capillary refill was normal. He was neurovascularly intact, with no focal neurological deficits, apart from known blindness. His skin was notable for bilateral, lower extremity ulcers with minor brown skin discoloration, but without redness or purulent discharge. He was alert and oriented to person, place, and time.

Given that the patient’s primary concern was dyspnea but with clear lung sounds on exam, a broad workup was performed to evaluate for possible causes. This included troponin to evaluate for acute coronary syndrome, brain naturetic peptide (BNP) for possible new-onset heart failure, and a D-dimer as we were considering a pulmonary embolism on the initial differential diagnosis. His initial electrocardiogram (ECG) showed normal sinus rhythm with no arrhythmia or evidence of ischemia.

*CPC-EM Capsule*

What do we already know about this clinical entity?

*Metformin toxicity is a rare but serious complication of metformin use that requires swift intervention to prevent severe complications*.

What makes this presentation of disease reportable?

*Our patient had no previous renal dysfunction or severe gastrointestinal symptoms, presenting only with dyspnea*.

What is the major learning point?

*Metformin toxicity should be considered in any patient with anion gap metabolic acidosis with no other clear cause even with no known renal dysfunction*.

How might this improve emergency medicine practice?

*This case demonstrates the importance of considering metformin toxicity for any patient taking the medication and to begin prompt treatment*.

His primary labs were significant for severe anion gap metabolic acidosis. His pH was 6.89 (reference range: 7.32–7.42); venous partial pressure of carbon dioxide, 9 mm Hg (42–55 mm Hg); venous bicarbonate level 2 mmol/L (23–33 mmol/L); and anion gap, 44 milliequivalents per liter (mEq/L) (4–12 mEq/L). He was hyperkalemic to 6.2 mmol/L (3.4–5.1 mmol/L) (Table 1). His kidney function was also found to be vastly reduced, with a creatinine of 11.25 mg/dL (0.70–1.30 mg/dL) and blood urea nitrogen level, 89 mg/dL (6–23 mg/dL).

Nephrology was emergently consulted, which recommended initiating fluid resuscitation, sodium bicarbonate, and furosemide, as well as calcium gluconate and insulin for hyperkalemia. The patient was given fluid resuscitation with 1 liter lactated Ringer’s; 150 mEq sodium bicarbonate; 40 mg intravenous (IV) Lasix push; 1 gram calcium gluconate; and 5 units IV regular insulin with one amp dextrose 50% to prevent hypoglycemia, as per hospital policy. Given his critically ill state, he was given 2 g of ceftriaxone in the ED. Of note, the troponin, BNP, and D-dimer that had been ordered as part of the initial dyspnea workup were all found to be elevated. However, given the patient’s severe metabolic acidosis, we believed the most likely cause for his dyspnea and tachypnea was respiratory compensation. Therefore, we deferred further cardiopulmonary evaluation to the inpatient team after the acidosis had been corrected. The patient was admitted to the intensive care unit (ICU) for further care following the return of the initial results.

On reassessment, we conducted a directed interview for potential causes of his anion gap metabolic acidosis. The patient specifically denied any ingestion of non-steroidal anti-inflammatories or acetaminophen, antifreeze, or ethanol. He reported a normal appetite and no unusual ingestions. He reported he had been taking furosemide, hydrochlorathiazide, and metformin as prescribed. His serum levels of salicylates and acetaminophen were undetectable. His secondary labs were significant for severely elevated lactic acid levels to 17.3 mmol/L (reference range: 0.5–2.0 mmol/L) and a minor beta-hydroxybutyrate elevation to 6.65 mmol/L (0.1–0.27 mmol/L) (Table 2), which led to the determination that this was primarily a lactic acid anion gap metabolic acidosis with minor ketoacidosis.

Once admitted to the medical ICU, the patient was started on emergent hemodialysis with norepinephrine vasopressor support (.05–0.1 micograms [mcg] per kilogram per minute for a mean arterial pressure goal > 65). Two days after admission, he was transferred to the floor. His workup revealed acute renal failure with unknown cause. Computed tomography of the abdomen and pelvis demonstrated severe right-sided hydronephrosis with ureteropelvic junction obstruction and severe parenchymal thinning. Urology deemed the ureteropelvic junction obstruction chronic due to the corresponding renal atrophy and a nuclear medicine evaluation showing 6% of renal function on the right. Urology consult did not feel these findings contributed to the patient’s acute presentation. His infectious workup returned with negative blood cultures at five days. Given this finding in combination with no infectious symptoms it was thought unlikely that sepsis contributed to the presentation. Nephrology consult favored the lactic acidosis to be metformin-associated lactic acidosis; however, the inciting renal insult remained unclear.

The patient underwent continuous renal replacement therapy for one night and then received one additional hemodialysis session two days later. He ultimately regained renal function before being discharged on day 10 of hospitalization with no need for outpatient dialysis. His metformin, hydrochlorathiazide, and furosemide were discontinued at time of discharge. The patient’s serum metformin levels resulted several weeks later, measuring to be 27 mcg/mL by high-performance liquid chromatography-tandem mass spectrometry. The reported therapeutic range was 1–2 mcg/mL. It was also reported that metformin-associated lactic acidosis was generally associated with plasma concentrations > 5 mcg/mL.

## DISCUSSION

Metformin toxicity is a rare but serious complication of metformin use with high morbidity and mortality. Since the 1970s when cases of metformin toxicity were first reported, the broad term metformin-associated lactic acidosis has been used to describe metformin toxicity. However, in 2017 it was suggested that this toxicity exists as a spectrum of diseases.^8^ This spectrum was developed to differentiate lactate accumulation due to metformin vs other causes vs a combination of the two.^9^

The spectrum of metformin toxicity has three subcategories of lactic acidosis. Metformin-induced lactic acidosis, metformin-associated lactic acidosis, and metformin-unrelated lactic acidosis. Metformin-induced lactic acidosis occurs when metformin is the cause of the patient’s illness. Patients will typically have metformin concentrations significantly greater than normal levels. This is commonly associated with an acute metformin overdose or a subacute accumulation of a standard metformin dose due to renal dysfunction.^10^ Metformin-unrelated lactic acidosis is on the opposite end of the spectrum, where patients who happen to be on metformin have another clear, underlying cause for their lactic acidosis (eg, sepsis, stroke, cardiogenic shock). The metformin levels of these patients, if measured, will be within normal range. Metformin-associated lactic acidosis lies between the two. This is thought to occur when a patient has an underlying illness, and metformin use amplifies the degree of lactic acidosis the patient experiences. While metformin levels will be elevated, generally they will not be as elevated as in cases of metformin-induced lactic acidosis.^8^,^10^

Differentiating between diseases on this spectrum in the ED is challenging, particularly between metformin-induced and metformin-associated lactic acidosis as serum metformin levels are not often readily available. It is, therefore, recommended that emergency clinicians treat as metformin-associated lactic acidosis, as this is the most severe form of metformin toxicity and has the highest associated mortality on the spectrum.^10^

Metformin-associated lactic acidosis is a rare but life-threatening cause of anion gap metabolic acidosis. It is estimated there are < 10 true events of metformin-associated lactic acidosis per 100,000 patient years of exposure.^6^,^7^ This means that while metformin-associated lactic acidosis should be on the differential for all patients taking metformin with severe anion gap metabolic acidosis, it is important to rule out other potential causes. Specifically, it is important to rule out glycol ingestion, acetaminophen overdose, methanol intake, aspirin overload, uremic renal failure, and ketosis.

Once a lactic acidosis has been identified, it can be classified as one of two types, A and B. Type A occurs from tissue hypoperfusion, which shifts cellular metabolism toward anaerobic glycolysis and results in increased lactic acid production. Type A includes shock states, regional ischemia, and cardiopulmonary arrest.^11^ Type B is due to overwhelming pyruvate levels and is the mechanism of action for metformin-associated lactic acidosis.^11^ Its specific mechanism includes the inhibition of complex one of the mitochondrial respiratory chain, which shifts the body toward anaerobic metabolism and increases lactic acid generation.^12^

Type A lactic acidosis due to infection or tissue ischemia was considered in this case. The source of the lactic acid seemed unlikely to be due to an acute ischemic event, as the patient had a normal ECG and no chest pain or abdominal pain, with a benign abdominal exam. It was similarly less likely to be from an infectious source, as he was afebrile and had no signs or symptoms of a focal infection. However, given limited information in the ED, the patient was given ceftriaxone, and blood cultures were obtained, as metformin-associated lactic acidosis often occurs in combination with sepsis. It is important to rule these sources of lactic acid out before attributing it to metformin, and in the ED setting it is important to cover the patient with antibiotics in case an occult infection is contributing to the presentation.

If other causes of lactic acidosis have been reasonably ruled out, the emergency clinician should suspect metformin-induced and metformin-associated lactic acidosis. It is essential to begin correcting the electrolyte imbalances as well as giving fluid resuscitation if no contraindications exist. Progression of acidosis can lead to conditions such as shock that further reduce lactate clearance, causing a positive feedback loop, which underlines the importance of early intervention. In our literature review, renal replacement therapy was associated with favorable outcomes even in patients with lower pH levels and higher lactate or metformin levels.^13^ Current literature also shows no correlation between plasma levels of metformin or lactate with early mortality.^14^

## LIMITATIONS

It should be noted that this patient’s workup and metformin levels could be argued to fit a diagnosis closer to metformin-induced rather than metformin-associated lactic acidosis. We chose the terminology metformin-associated lactic acidosis based on our working ED diagnosis, and the suspected diagnosis throughout the patient’s hospital stay. Nephrology classified the patient as likely metformin-associated lactic acidosis at the time of discharge; however, the significantly elevated metformin levels that later returned indicate our patient’s condition could have been classified as metformin-induced lactic acidosis. The patient had no history of metformin overdose or renal dysfunction; however, given the mortality associated with metformin-associated lactic acidosis it was treated as such in the ED. Our case shows that the distinction between diseases on the metformin toxicity spectrum can be nearly impossible to determine while hospitalized; however, resuscitation and treatment in the ED remains the same.

## CONCLUSION

Metformin toxicity is an anion gap metabolic acidosis secondary to elevated lactate levels in patients taking metformin. It classically presents with GI symptoms in a patient with a history of renal disease; however, as this case demonstrates, it is important to maintain metformin toxicity on the differential diagnosis for patients taking metformin who present with shortness of breath and a lactic acidosis without an alternate identifiable etiology. Although metformin levels will not be confirmed until long after the patient has left the ED, excluding other potential causes of lactic acidosis creates a reasonable suspicion for metformin-induced or metformin-associated lactic acidosis in patients on metformin. The emergency physician has an essential role in this workup and is positioned to expedite proper treatment.

## Figures and Tables

**Table 1:** Initial laboratory workup of a 68-year-old male diagnosed with metformin-associated lactic acidosis.

Lab	Value	Reference Interval
PH, venous	6.86	7.32–7.42
PH CO_2_, venous (mm Hg)	9	42–55
PH O_2_, venous (mm Hg)	78	25–40
HCO_3_, venous (mmol/L)	2	23–33
Base excess, venous (mmol/L)	−29.3	−2.0–2.0
WBC (10e3/μL)	10.5	4.0–10.0
RBC (10e6/μL)	3.6	4.4–5.9
Hemoglobin (g/dL)	12.3	13.7–17.5
Mean corpuscular volume (fL)	110	79–98
Platelet count (10e3/μL)	155	160–400
Neutrophil %	86	43–74
Lymphocyte %	8	17–46
Monocyte %	3	4–13
Eosinophil %	0	0–6
Basophil %	1	0–1
Blood urea nitrogen (mg/dL)	89	6–23
Sodium (mmol/L)	138	136–145
Potassium (mmol/L)	6.2	3.4–5.1
Chloride (mmol/L)	92	98–107
CO_2_, total (mmol/L)	2	22–29
Anion gap (mmol/L)	44	7–15
Glucose (mg/dL)	121	70–180
Creatinine (mg/dL)	11.25	0.70–1.30
eGFR (mL/min/1.73m2)	4	≥ 60
Albumin (g/dL)	4.2	3.8–5.0
Protein, total (g/dL)	7.2	6.1–8.2
Alkaline phosphatase (U/L)	101	40–129
AST (U/L)	24	< 50
ALT (U/L)	16	< 42
Bilirubin, total (mg/dL)	0.3	0.2–1.2
Troponin (ng/L)	100	≤ 16
D-dimer (mg/L FEU)	2.24	0.0–0.50
BNP (pg/mL)	3,232	≤ 125

*AST*, aspartate aminotransferase; *ALT*, alanine transaminase*; BNP*, brain naturetic peptide*; CO_2_*, carbon dioxide*; dL*, deciliter; *eGFR*, estimated glomerular filtration rate; FEU, fibrinogen equivalent unit; *g*, gram; *L*, liter; *10e3*, 10,000*; uL*, microliter; μ*10e6*, 1 million; *mg*, milligram; *mmol*, millimole*; ng*, nanogram; *pg*, picogram; *RBC*, red blood cell count; *WBC*, white blood cell count.

**Table 2:** Secondary laboratory workup of 68-year-old male diagnosed with metformin-associated lactic acidosis.

Lab	Value	Reference Interval
Acetone (mg/dL)	Not detected	Not detected
Ethanol, serum (g/dL)	Not detected	Not detected
Isopropanol (mg/dL)	Not detected	Not detected
Methanol (mg/dL)	Not detected	Not detected
Ethylene glycol (mg/dL)	Not detected	Not detected
Salicylate (mg/dL)	< 0.3	≤ 29.9
Acetaminophen (mg/L)	< 5	≤ 30
Beta-hydroxybutyrate (mmol/L)	6.65	0.1–0.27
Lactic acid (mmol/L)	17.3	0.5–2.0
Blood cultures	In process	No growth detected
Metformin (mcg/mL)	In process	1–2

*dL*, deciliter; *g*, grams; *mg*, milligram; *mmol*, millimoles; *mcg*, microgram.
